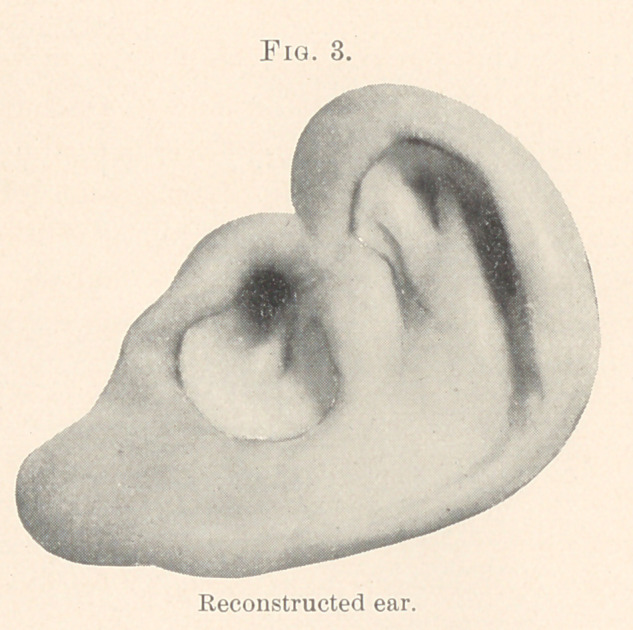# The Preparation of the Mouth for an Artificial Denture Preparatory to Taking an Impression

**Published:** 1898-05

**Authors:** Robert H. Nones

**Affiliations:** Philadelphia


### THE PREPARATION OF THE MOUTH FOR AN ARTIFICIAL DENTURE PREPARATORY TO TAKING AN IMPRESSION.[1]

BY ROBERT H. NONES, D.D.S., PHILADELPHIA.

In offering, this evening, this paper, it is not with the idea of presenting any originality whatever, but rather of calling attention to a part of that branch of dentistry which is seemingly much neglected,—namely, prosthesis.

---

[1] Read at a meeting of the Academy of Stomatology, January 25, 1898.

And why neglected? Surely not because it is so thoroughly understood, nor because it is of little importance; certainly not the latter, for the last resort of anything must naturally be of vast importance. Prosthesis is the last resort in dentistry, a reliance when our patients have arrived at the stage when we have exhausted all means for preservation of the natural organs.

In presenting the paper it is with the idea that a discussion and a line of thought may be brought out which will be of interest to perhaps not only a few, but many of us.

Naturally the first step in the preparation of the mouth for an artificial denture preparatory to taking an impression would be an examination of the mouth, upon the thoroughness of which depends largely the success or failure. We must observe carefully the condition of the parts, whether normal or abnormal; discover, if possible, the cause or causes of the abnormal conditions, and remove or treat them. We must give particular attention to the extraction or retention of teeth or roots, and at the same time plan the future denture, mapping out what to make and how to make it.

In the examination of an edentulous mouth notice the general shape, whether it be regular or irregular, the vault high or low, the ridge prominent or flat. Note carefully any hard or bony protuberances in the vault of the palate, as well as an irregularly resorbed ridge of either the upper or lower jaw.

If soft or flabby conditions of the tissues exist, they must be equally well noticed that proper relief or pressure may be given with the denture, or that they may receive treatment either surgically or medicinally. Location of the muscles must be carefully observed, as it is of vital importance to the proper outlining of the plate, attention to which gives comfort to the patient. All these conditions have considerable bearing on the planning of the denture and must be carefully observed.

Another equally important condition is that of a fissure of the vault, starting in the soft palate and extending at times quite far into the hard palate. This is often so narrow in appearance as to deceive one as to its depth, except upon close examination. These fissures run usually through the centre of the mouth, but may be found double, one on either side of the hard central prominence.

Frequently unsuccessful dentures would have been successful ones had proper cognizance been taken of this condition. The knowledge of its existence will enable us to avoid a space between the denture and the mouth, and to exclude the air which would otherwise enter.

Inflamed or irritated mouths, referring for the present to edentulous mouths, may result from various causes, both local and systemic.

Dyspepsia plays an important part, and its treatment would naturally be relieved by systemic treatment at the hands of the general practitioner.

The immoderate use of ardent spirituous liquors and of tobacco may also cause conditions of irritability. Upon the partial or total cessation of the abuses we should naturally expect relief.

The most frequent cause of these inflamed or irritated conditions, and one which should be looked for, is a previously worn denture. How frequently will a patient, when we are about to make an examination, quickly remove from the mouth a denture, and with an expression of shame carefully close it from view in a napkin or in a handkerchief. The cause of the embarrassment, as a rule, is the condition of the denture, which in many instances is covered with particles of food from the previous meal or meals, and for which generally profuse apologies are offered.

Another cause, less frequent, but not at all infrequent, is the adaptation of foreign substances, such as wax, cotton, and even chewing-gum to ill-fitting dentures. In this connection I recall an incident showing apparently how an otherwise cleanly person may be unconscious of the actual condition of the mouth.

A gentleman, about sixty years of age, his general appearance denoting refinement, neatness, and cleanliness, sought my services to have a partial lower metal clasp piece repaired. One of the natural teeth had become quite loose, and he wished an artificial one attached to the plate in its stead. On examination I noticed it was rather a difficult piece to remove, and told him to remove it, as I wished to save him the inconvenience which is often occasioned by the removal by the dentist of such a piece. I shall never forget his look of mingled surprise and helplessness when he told me it had never been removed from the time it was first inserted, some five years previously.

I removed the piece with some difficulty, making no effort to hurry it past his nostrils during the withdrawal from the mouth, which caused him, as I had anticipated, to withdraw his head. In condition it was, almost needless to remark, foul and covered with calculus and *débris*. The discomfiture of the patient was marked and not at all feigned. He informed me that he always believed himself to be cleanly, bathing at least once daily and changing his under-garments as precisely. His mortification was intense.

While we should naturally expect human beings to be cleaner in the mouth than any other part, it is our duty always to instruct them in regard to the proper care and removal of dentures. We cannot expect to find a mouth in other than an abnormal condition under such circumstances.

Frequently we find the mouth irritated from roughness on the plate. In some cases there are prominences upon so-called temporary plates which were formerly seated in tooth-sockets; in other cases, sharp, rough, angularly outlined, and deep vacuum-chambers, or perhaps too highly outlined margins which impinge upon the muscles and mucous membrane, cutting them and creating irritable wounds; this occurs markedly in lower dentures. How can we possibly expect success to follow the making of a denture on such faulty foundations, and yet how frequently are impressions taken under such conditions, success being expected to follow, and when it does not, this branch of dentistry is condemned and detested. It is attention to such matters that raise prosthetic dentistry from a mechanical to a scientific basis, and tends to bring pleasure in the work as well as satisfactory results.

The treatment in such cases as the foregoing is simple. First comes the removal of the cause or causes. If the denture be a metal one, carefully boil it in a dilute solution of sulphuric acid; upon removal, allow it to cool, when it should be washed with soap and water, and placed in a solution of sodium bicarbonate, thus neutralizing any acid beneath the porcelain teeth, after which any roughness should be removed and sharp or rough edges of vacuum-chamber dressed down and smoothed. The plate should then be brushed with a stiff lathe-brush and pumice, then polished and carefully washed.

The treatment of a vulcanite denture is the same, with the exception that it is allowed to stand in a cool solution of hydrogen dioxide in place of warm sulphuric acid.

Small pin-point prominences may be looked for, caused by the vulcanite forcing into the pores or small bubble-like places on the surface of the plaster, these are frequently a source of great irritation and annoyance to a patient, and they can be largely avoided by using a tin model instead of a plaster one. When they exist in a plate they should be removed.

The denture after proper treatment, providing it fits well enough and is not likely to cause further irritation, may be worn until the mouth is in condition for the impression, otherwise its use must be discontinued.

The medicinal treatment of the effects produced by the roughness consists in the application of antiseptic mouth-washes, such as "listerine," "borine," etc. The most effectual and quickest treatment I have found is the painting of the inflamed parts with tincture of iodine, together with the frequent use of a three-per-cent. solution of hydrogen dioxide by the patient as a mouth-wash.

During the examination of the mouth not infrequently will be found prominent points of the alveolar process, due to uneven resorption after the extraction of the teeth. These points should be removed by surgical means.

The attachment of the frænum of the lip to the alveolar process frequently being very low, it may interfere with the upper margin of the denture, or we may find what is known as a double lip or a false lip, which is caused by an ill-fitting plate or one extending too high. In some cases this extends below the natural lip. These with the excessively flabby conditions may receive surgical treatment.

What is of less frequent occurrence is the prominence of the alveolar process, which may be so excessive as to eliminate the idea of an artificial denture. I recall such a marked case, which I was fortunate enough to see both before and after operation for the condition. This was performed by Dr. M. H. Cryer, who kindly gave me the opportunity to see the case. Any one upon looking at the patient with her lips closed, which was a difficult act for her to perform, would have concluded that her dentist had made a grave mistake in the fulness of her denture. Dr. Cryer performed an operation, removing about a quarter of an inch of the labial and buccal process, thus placing the mouth in a fit condition for an artificial denture. The surgical aspect of this subject I note not alone on account of its great importance, but to show how an effect may be created, if we are aware of the proper course to pursue.

Should the mouth not be an edentulous one, we may look for other causes of irritation besides those already noted.

One, and an important one, is salivary calculus, an irritant which should generally be removed. With exceptions to this rule I shall deal later. The methods of removal are so well known that it is unnecessary to repeat them here. I have been very successful with that pursued and I believe suggested by Dr. Henry Register, the use of iodine in connection with the ordinary scaling method, which I follow up with and have the patient use freely the hydrogen dioxide. In fact, hydrogen dioxide will keep a mouth in better condition than anything else I have tried, and I give my patients to understand that they cannot use too much of it.

In mouths containing some natural teeth we will frequently find partial dentures creating irritation of the gums about the necks of the teeth. A sinking in of the plate and a bulging out of the gum around and between the teeth and plate is caused most frequently by the plate not fitting accurately to the natural teeth. We may also look for the same effect from roughness and uncleanliness, as in the case of full dentures, and they should be treated in like manner.

The extraction or retention of teeth or roots depends upon the advantages to be gained thereby. When they cannot be brought to a healthy state they are to be extracted. To make a broad assertion, they are not to be extracted unless positively detrimental to the health of the patient and to the success of the denture. We must remember that an artificial tooth is, as a rule, never as valuable as the natural organ. No tooth is to be extracted merely to facilitate the taking of an impression or the making of a denture; such action would be malpractice. We should adapt ourselves to proper circumstances rather than adapt the circumstances to ourselves.

Taking in regular order the teeth of the superior jaw and commencing at the centrals, if one or both are alone remaining, it is preferable to extract, on general principles; but there are exceptions, with which I wish to deal later.

I should extract first, on account of the difficulty of matching the natural teeth with porcelain; secondly, if left in, there will in all probability be interference with the bite, which, as a rule, is very short, thus making a very weak denture, from which the teeth are constantly being broken off; thirdly, breaks are liable to occur through the centre of the plate, which is much weakened by loss of continuity, although this, of a lesser evil, can be obviated by strengthening the plate; and fourthly, the forcing of the dentine against the centrals would be a constant source of irritation to them, causing resorption of the process supporting them, while the gum about the necks of the teeth would be continually irritated.

The laterals, if standing alone, should be extracted for the same reasons.

If all the four superior incisors are in place, they should be retained, other conditons permitting, as the cuspids can be artistically matched to them.

The cuspids should be kept whenever it is possible; even the roots should be preserved and crowned, as they not only retain that facial expression which it is almost impossible to regain with an artificial denture, but also guide and stay the bite.

They should be extracted, however, when they cannot be brought to a healthy condition, or when so abnormally situated as to interfere with proper adjustment of a denture, as, for instance, when interfering with the bite or protruding to such a degree as to cause disfigurement, or if drooping inward, especially if occluding inside of the lower teeth; or if there be excessive recession of the gum, or a tilting from or towards the median line, thus making it impossible to properly adjust artificial teeth between the natural teeth; or if out of line from or towards the centre, not allowing sufficient space for the proper size or number of teeth; or if elongated, giving a marked canine expression to the face.

All the foregoing conditions of the cuspids are detrimental to the proposed denture, and unless they can be corrected by the removal of the natural crowns and the adjustment of artificial ones, thus preserving the desired expression with the roots, the teeth should be extracted.

Single cuspids should be extracted, except when male patients of middle age seem loath to lose them because of good service. Smokers especially are apt to find such teeth serviceable in holding the cigar or pipe.

Bicuspids, except when on both sides of the mouth and useful as clasp attachments, should be extracted.

Molars should be retained whenever possible, as they afford a means of support by clasping in mouths which are difficult to fit with vacuum plates. The roots should be preserved and crowned for clasp attachment.

In the lower jaw none of the teeth should be extracted when it is possible to retain them, with possibly the exception of a single central or lateral or laterals in pairs.

The other teeth afford a means of support for a denture until such time as the patient may become accustomed to its management.

In extraction all sharp prominences of the alveolar process should be removed, thus permitting a smooth, even resorption.

With aged or invalid patients it seems occasionally to be better to allow quite large deposits of salivary calculus to remain upon the teeth, for the reason that were it at once removed and a plate made it is likely that the teeth would be promptly lost and a new denture necessitated. By leaving the calculus the patient becomes accustomed to the use of the denture, and many changes afterwards necessitated are more readily tolerated.

In this connection I recall a case in my own practice which will probably illustrate my meaning.

Fig. 1.

Mutilated ear.

Fig. 2.

Artificial vulcanite piece.

Fig. 3.

Reconstructed ear.

Some three or four years ago I made a partial upper gold clasp piece for a very amiable lady. If I remember correctly, there were several bicuspid and molar roots on each side of the mouth, which I wished to extract. Upon so informing the patient, she asked, "Can you not let them remain? They give me no trouble, and will probably outlast me." I read in her look that she was an invalid, and afterwards one of her friends informed me that she was sick, but did not know her trouble, nor ever mentioned her illness when among her friends. I adjusted the piece over the roots, and it was satisfactory. She died about two months ago, and upon post-mortem examination cancer of the stomach was revealed. Of course, it was a source of satisfaction to know that I adapted myself to the circumstances rather than the circumstances to myself.

The preparation of the mouth for a denture or other forms of prosthesis may be as scientifically carried out as any other part of dentistry. Prosthetic dentistry is what we make it.

This recalls to my mind the answer of the celebrated Joshua Reynolds. When asked as to how he mixed his colors, he replied, "With brains."

## Figures and Tables

**Fig. 1. f1:**
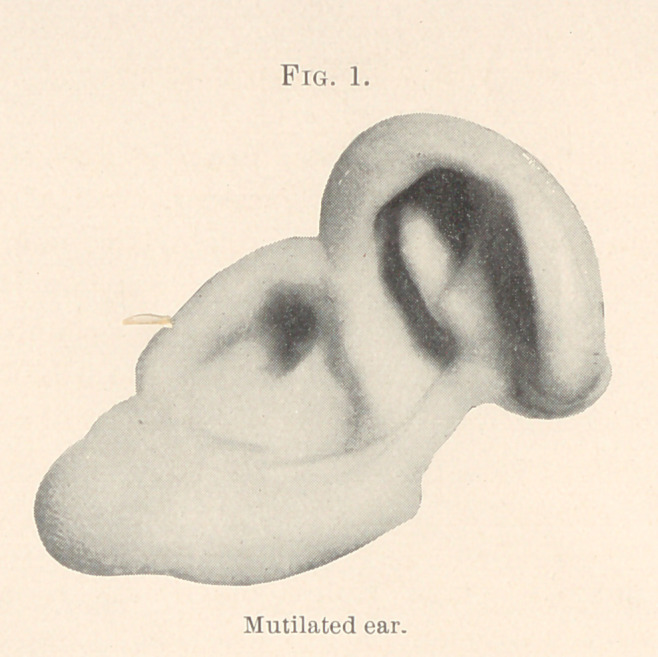


**Fig. 2. f2:**
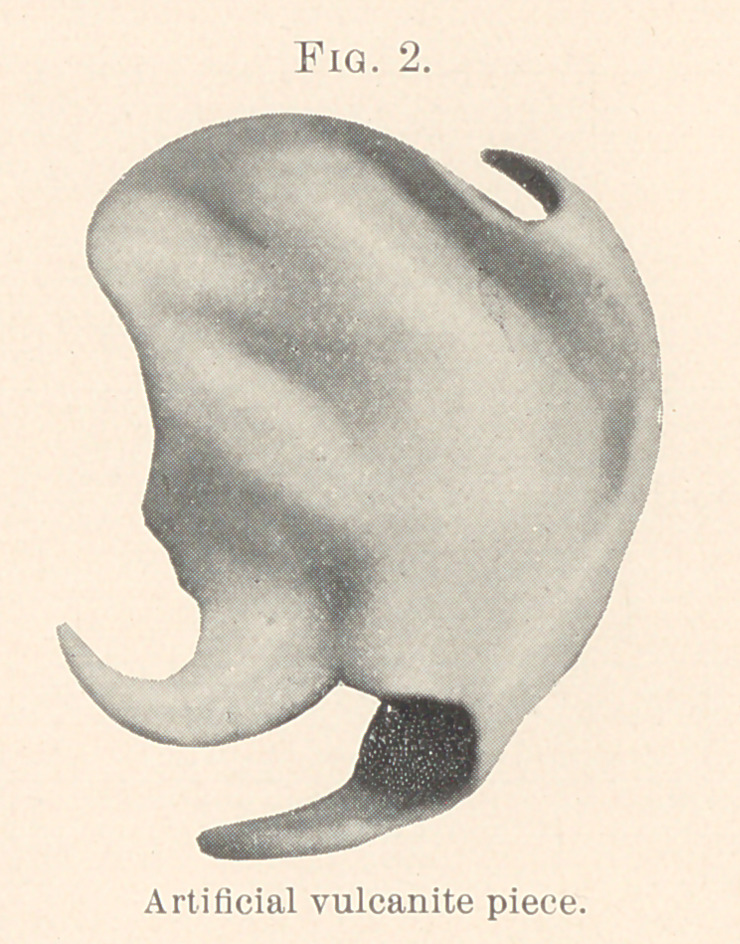


**Fig. 3. f3:**